# Prognostic value of the systemic immune-inflammation index in acute limb ischemia undergoing endovascular therapy

**DOI:** 10.3389/fcvm.2026.1732152

**Published:** 2026-08-12

**Authors:** Shuji Morikawa, Nao Takahashi, Ryo Ohinata, Takashi Mishina, Hikaru Hayakawa, Yuki Kumihashi, Naoya Inoue, Yohei Takayama, Toyoaki Murohara

**Affiliations:** 1 Department of Cardiology, Chutoen General Medical Center, Kakegawa, Shizuoka, Japan; 2 Department of Cardiology, Nagoya University Graduate School of Medicine, Nagoya, Japan

**Keywords:** acute limb ischemia, endovascular therapy, inflammation, prognosis, systemic immune-inflammation index

## Abstract

**Background:**

Acute limb ischemia (ALI) is a vascular emergency associated with major amputation and mortality, highlighting the need for early risk stratification. The systemic immune-inflammation index (SII), calculated from platelet, neutrophil, and lymphocyte counts, is a composite biomarker reflecting systemic inflammation and immune status. Although SII has demonstrated prognostic value in various cardiovascular diseases, its role in patients with ALI undergoing endovascular therapy remains unclear.

**Objective:**

To evaluate the association between baseline SII and 12-month outcomes in patients with ALI undergoing endovascular therapy.

**Methods:**

This retrospective, single-center cohort study included 47 consecutive patients with ALI treated with endovascular therapy. Patients were dichotomized at the median baseline SII into high and low SII groups. The primary outcome was a composite of major amputation or all-cause mortality within 12 months. Kaplan–Meier survival analysis and multivariable Cox proportional hazards regression models were performed to assess associations between baseline SII and outcomes, adjusting for age, sex, severe ischemia (Rutherford IIb–III), and renal function (eGFR). Receiver operating characteristic analysis evaluated the discriminatory ability of SII.

**Results:**

The primary outcome occurred in 20 patients (42.6%) and was more frequent in the high-SII group than in the low-SII group (62.5% vs. 21.7%, *P* = 0.008). Kaplan–Meier analysis demonstrated a higher event rate in the high-SII group (log-rank test, *P* = 0.002). In an exploratory multivariable Cox model, older age and high SII were associated with the primary composite outcome, although the estimate for high SII was imprecise with a wide confidence interval. Receiver operating characteristic analysis showed an area under the curve of 0.81 for SII, and exploratory comparisons did not demonstrate superiority of SII over the neutrophil-to-lymphocyte ratio, platelet-to-lymphocyte ratio, or C-reactive protein.

**Conclusion:**

In this exploratory cohort study, elevated baseline SII was associated with a higher risk of the composite outcome of major amputation or all-cause mortality within 12 months in patients with ALI undergoing endovascular therapy. This association appeared to reflect overall adverse prognosis, whereas its specific association with major amputation remains uncertain. These findings, including the ROC-based analyses, should be considered hypothesis-generating and require validation in larger prospective multicenter studies.

## 1 Introduction

Acute limb ischemia (ALI) is a severe vascular emergency characterized by a sudden reduction in limb perfusion that threatens limb viability and can rapidly lead to major amputation or death if not promptly treated (1, 2). Despite advances in surgical and endovascular therapies (3–5), outcomes remain poor, with reported mortality rates of approximately 15%–30% and major amputation rates of approximately 10%–20% in patients with ALI (3–5). These findings underscore the urgent need for reliable markers that support early risk stratification for adverse outcomes in this population.

Systemic inflammation plays a central role in the pathophysiology of ALI, contributing to endothelial dysfunction, thrombosis, and ischemia–reperfusion injury (6). In both acute and chronic vascular diseases, such as ALI and peripheral artery disease, traditional inflammatory markers including C-reactive protein (CRP) and leukocyte count are commonly used to assess systemic inflammatory status (7). However, the ability of these markers to support long-term risk stratification, including mortality and major amputation, remains limited, which has prompted growing interest in more comprehensive inflammatory indices (8). Moreover, few laboratory inflammatory markers have been established for long-term prognostic risk assessment in patients with acute limb ischemia. The systemic immune-inflammation index (SII), calculated as (platelet count × neutrophil count) ÷ lymphocyte count, has recently gained attention as a composite inflammatory index reflecting the interplay between innate immune activation and systemic inflammation (9). Initially introduced in oncology (9) and subsequently validated in cancer populations (10), the SII has been associated with outcomes in several cardiovascular conditions and clinical settings, including carotid artery stenting, coronary artery disease, and heart failure (11, 12). Given its simplicity and incorporation of multiple immune components, the SII may reflect multiple aspects of systemic inflammation.

Given the pronounced inflammatory response observed in ALI (13), the SII may provide insights into disease severity and long-term clinical outcomes in affected patients. However, the association between SII and outcomes in ALI has not yet been well established. In particular, the association between SII and long-term clinical outcomes in patients undergoing endovascular therapy for ALI remains insufficiently investigated. To date, very few studies have examined the association between the baseline SII and long-term outcomes such as major amputation and all-cause mortality in this setting. Therefore, we conducted this study to evaluate the association between baseline SII and 12-month outcomes in patients with ALI, hypothesizing that an elevated SII is associated with a higher likelihood of major amputation or all-cause mortality within 12 months.

## 2 Methods

### 2.1 Study design and patient population

This retrospective, single-center cohort study was conducted at Chutoen General Medical Center, a regional tertiary care facility in Japan. Medical records of consecutive patients diagnosed with ALI who underwent endovascular therapy and were admitted between June 2013 and April 2024 were retrospectively reviewed. ALI was defined according to the Rutherford classification, which characterizes a sudden decrease in limb perfusion that threatens limb viability (2). A total of 56 patients initially met the inclusion criteria.

**Figure 1:**
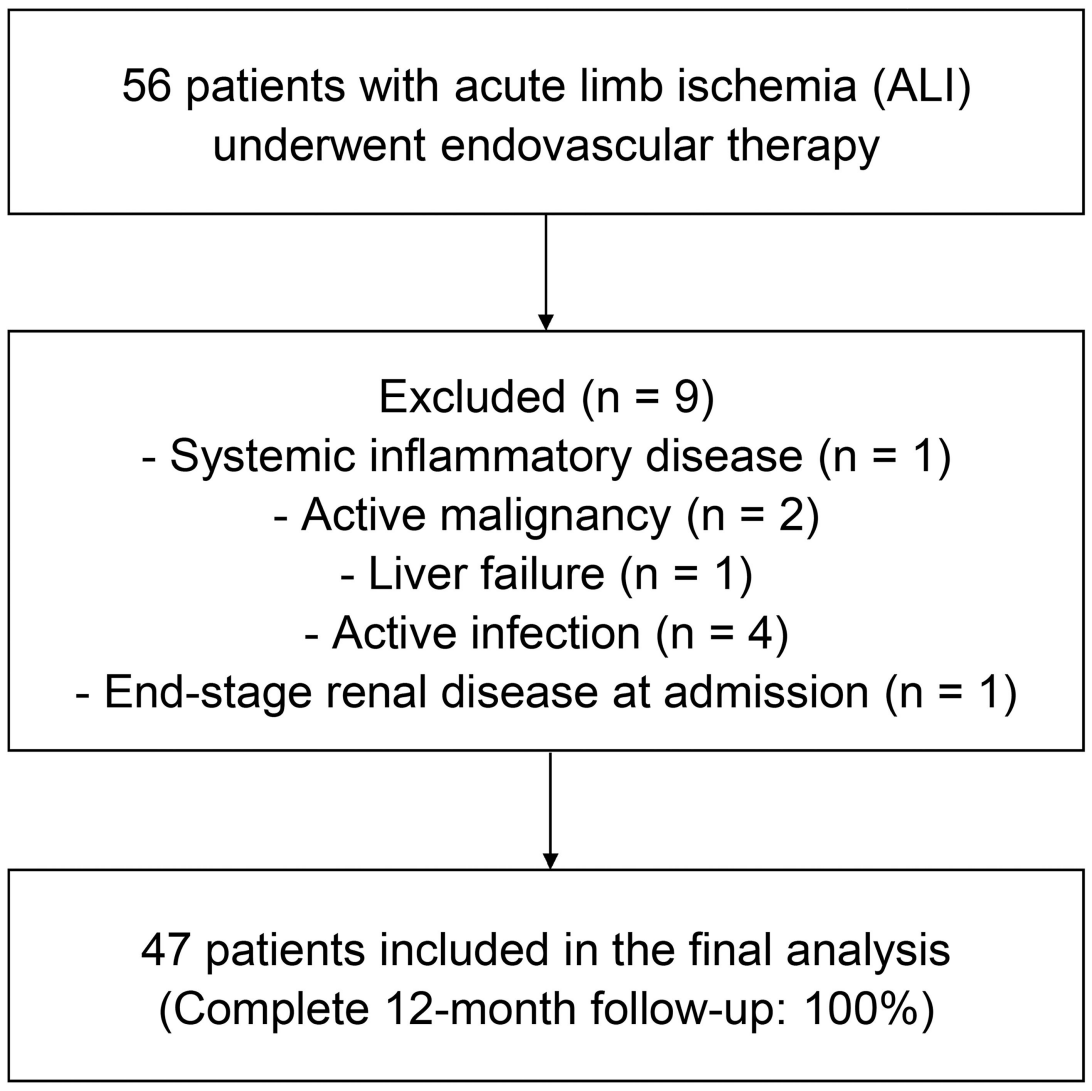
Flow diagram of patient inclusion, exclusion, and follow-up.

To minimize confounding factors that could affect systemic inflammatory markers such as the SII, the following exclusion criteria were applied: (1) systemic inflammatory diseases; (2) active malignancies; (3) liver failure; (4) active infections; and (5) end-stage renal disease at admission. Active infection was defined as clinically suspected or documented infection requiring antimicrobial therapy at admission or infectious symptoms supported by laboratory or imaging findings. Systemic inflammatory disease was defined as a documented chronic autoimmune or systemic inflammatory disorder requiring ongoing treatment or considered clinically active at admission. After applying these exclusions, 47 patients were included in the final analysis (Figure 1). No formal sample size calculation or *a priori* power calculation was performed because of the retrospective and exploratory nature of the study. All consecutive patients who met the eligibility criteria during the study period were included.

Baseline demographic, clinical, and laboratory data were collected, including age, sex, comorbidities, Rutherford classification, and initial neutrophil, lymphocyte, and platelet counts. Lesion location was assessed based on angiographic findings and categorized according to the presence or absence of aorto-iliac involvement as an indicator of proximal lesion involvement. Blood samples for laboratory measurements used to calculate the SII were obtained at hospital admission as part of the initial clinical evaluation before endovascular intervention and routine anticoagulation therapy, and hematological parameters were analyzed using standard laboratory methods. The SII was calculated as platelet count multiplied by neutrophil count divided by lymphocyte count (9). $SII=\frac{\;platelet\;count\;\times \;neutrophil\;count}{lymphocyte\;count}$

For descriptive comparisons, patients were divided into high and low SII groups based on the median SII value (834). In addition, the SII was analyzed as a continuous variable in multivariable models to evaluate its association with outcomes.

The severity of ischemia was categorized according to the Rutherford classification, with categories IIb and III defined as “severe ischemia,” as previously described (2). The study was approved by the Ethics Committee of Chutoen General Medical Center (approval number: 1319250908) and conducted in accordance with the principles of the Declaration of Helsinki. The requirement for informed consent was waived by the Institutional Review Board owing to the retrospective nature of the study.

### 2.2 Procedure

ALI diagnosis was established based on clinical symptoms and imaging findings (contrast-enhanced computed tomography or duplex ultrasonography). Endovascular interventions—including balloon angioplasty, stent placement, and aspiration thrombectomy—were selected according to the occlusion site and the severity of ischemia. Treatment decisions were made at the discretion of the attending physician, and antithrombotic therapy was administered in accordance with institutional protocols. Systemic anticoagulation with intravenous unfractionated heparin was routinely initiated at the time of diagnosis unless contraindicated. Additional heparin was administered during endovascular procedures as appropriate according to institutional practice. Electrocardiography and transthoracic echocardiography were routinely performed as part of the clinical evaluation to assess potential cardiac sources of embolism. Final below-the-ankle perfusion was assessed on the completion angiogram after endovascular treatment. Below-the-ankle perfusion was considered present when contrast opacification of vessels distal to the ankle was visible on final angiography and absent when no visible distal contrast opacification was observed. This was a qualitative binary angiographic assessment, and no formal quantitative below-the-ankle perfusion scoring system or independent core laboratory assessment was used. Post-procedural antithrombotic therapy was individualized after the index procedure. Anticoagulation was continued or initiated when clinically indicated, such as in patients with atrial fibrillation or suspected embolic sources, whereas antiplatelet therapy was used according to the revascularization strategy and device type. The final regimen was determined by embolic risk, bleeding risk, and the underlying clinical condition. Ischemia severity was assessed using the Rutherford classification; however, detailed information regarding ischemia duration, embolic versus thrombotic etiology, and quantitative thrombus burden was not consistently available and therefore was not included in the analysis. Unplanned reintervention during follow-up was reviewed from medical records when documented; however, because it was a post-procedural time-dependent event and the number of outcome events was limited, it was not included as a covariate in the primary multivariable model. The date of major amputation was recorded based on medical record review.

### 2.3 Outcomes

The primary outcome was a composite of major amputation (defined as amputation at or above the ankle) or all-cause mortality within 12 months after admission. This composite endpoint was selected because both major amputation and death represent clinically severe adverse outcomes after acute limb ischemia and together reflect overall adverse prognosis in this high-risk population. Given the limited sample size and number of outcome events, the composite endpoint was also used to preserve statistical power for the primary analysis. Secondary outcomes included major amputation and all-cause mortality analyzed separately as individual components of the primary composite outcome to facilitate interpretation of the individual clinical events.

Follow-up information was obtained through reviews of hospital medical records and, for patients who could not be followed within the hospital system, through structured telephone interviews.

### 2.4 Statistical analysis

Continuous variables with a normal distribution were expressed as means ± standard deviations and compared using Student's *t*-test. Non-normally distributed variables, including D-dimer, CRP, and serum creatinine, were expressed as medians (interquartile range) and compared using the Mann–Whitney U test. D-dimer values were unavailable for three patients due to missing measurements at admission; therefore, these patients were included in all other analyses but excluded only from comparisons involving D-dimer levels (available-case analysis). No other variables had missing data. Categorical variables were summarized as numbers (percentages) and compared using the chi-square test or Fisher's exact test, as appropriate.

Event-free survival was estimated using the Kaplan–Meier method and compared between the high and low SII groups using the log-rank test. For the primary composite outcome, the event time was defined as the time to the first occurrence of either major amputation or all-cause death; patients without either event were censored at the last follow-up. Multivariable Cox proportional hazards models were constructed to evaluate factors associated with the primary outcome. Covariates included age, sex, severe ischemia (Rutherford IIb–III), estimated glomerular filtration rate (eGFR) calculated using the Japanese Society of Nephrology equation, and SII. These covariates were selected based on prior evidence, clinical relevance, and data availability. Severe ischemia and renal function (as assessed by eGFR) were included as covariates based on previous studies demonstrating their association with poor prognosis in patients with ALI (3, 14). Because of the limited number of outcome events in this study, the number of covariates included in the multivariable model was intentionally restricted to avoid model overfitting. The multivariable Cox models were considered exploratory, and effect estimates were interpreted cautiously with attention to statistical precision, including the width of the confidence intervals.

As an additional *post hoc* exploratory analysis addressing the potential relationship between SII and ischemia severity, SII values were compared between patients with severe ischemia, defined as Rutherford IIb–III, and those without severe ischemia, defined as Rutherford I–IIa, using the Mann–Whitney U test. We also descriptively evaluated the incidence of the primary composite outcome according to SII category within ischemia-severity strata. Because of the small number of patients and events in each stratum, these stratified analyses were considered exploratory and descriptive, and no definitive conclusions were drawn from these comparisons.

Sensitivity analyses were performed to assess the robustness of the main findings. First, the SII was analyzed as a continuous variable in the same multivariable Cox regression model adjusted for age, sex, Rutherford classification, and eGFR. Second, the SII was dichotomized using the ROC-derived cutoff value obtained from receiver operating characteristic (ROC) curve analysis, and the multivariable analysis was repeated using this cutoff. Quartile-based analysis was not conducted because of the limited sample size. These analyses were performed to assess whether the results were dependent on the specific definition of SII categories. The proportional hazards assumption was verified using Schoenfeld residuals.

ROC curve analysis was also performed to evaluate the discriminatory ability of the SII in relation to the primary composite outcome. The area under the curve, sensitivity, specificity, and ROC-derived cutoff value were calculated, with the cutoff determined by maximizing the Youden index. Additional exploratory analyses were performed to compare the discriminatory ability of SII with the neutrophil-to-lymphocyte ratio (NLR), the platelet-to-lymphocyte ratio (PLR), and CRP for the primary composite outcome. NLR and PLR were calculated as neutrophil count divided by lymphocyte count and platelet count divided by lymphocyte count, respectively. The AUCs were compared using an overall test for equality of ROC areas. Spearman's rank correlation analysis was performed to assess the association between SII and CRP.

All statistical analyses were performed using Stata version 19 (StataCorp LLC, College Station, TX, USA).

## 3 Results

### 3.1 Patient characteristics

A total of 47 patients with ALI were included in the final analysis. The median follow-up period was 12 months, and all patients completed the follow-up. The median SII was 834, and patients were divided into high (*n* = 24) and low (*n* = 23) SII groups based on the median value. The median SII values were 1988 (IQR, 1374–2922) in the high-SII group and 499 (IQR, 270–652) in the low-SII group. Baseline characteristics are summarized in Table 1.

**Table 1:** Baseline characteristics stratified by median SII value.

Variable	High SII (*n* = 24)	Low SII (*n* = 23)	*P*-value
Demographics			
Age, years (mean ± SD)	81 ± 9	77 ± 11	0.15
Male sex, *n* (%)	8 (33.3)	16 (69.6)	0.02
Comorbidities, *n* (%)			
Hypertension	17 (70.8)	17 (73.9)	0.81
Hyperlipidemia	6 (25.0)	6 (26.1)	0.93
Diabetes mellitus	7 (29.2)	4 (17.4)	0.49
Atrial fibrillation	13 (54.2)	12 (52.2)	0.89
Ischemia severity, *n* (%)			
Rutherford class I	1 (4.2)	5 (21.7)	0.097
Rutherford class IIa	3 (12.5)	8 (34.8)	0.093
Rutherford class IIb	18 (75.0)	10 (43.5)	0.028
Rutherford class III	2 (8.3)	0 (0)	0.49
Rutherford class IIb–III	20 (83.3)	10 (43.5)	0.004
Lesion location, *n* (%)			
Aorto-iliac involvement	5 (20.8)	7 (30.4)	0.45
Medications at admission, *n* (%)			
Aspirin	1 (4.2)	4 (17.4)	0.19
P2Y12 inhibitor	3 (12.5)	0 (0)	0.23
Anticoagulant	3 (12.5)	2 (8.7)	1.0
Statin	6 (25.0)	5 (21.7)	0.79
Laboratory data			
Albumin, g/dL	3.01 ± 0.68	3.43 ± 0.83	0.06
Creatinine, mg/dL	0.98 (0.54–1.68)	0.87 (0.75–1.04)	0.66
eGFR, mL/min/1.73 m^2^	57.2 ± 35.9	63.5 ± 26.2	0.50
C-reactive protein, mg/dL	1.88 (0.58–5.59)	0.49 (0.22–1.04)	0.028
White blood cell count/μL	9,008 ± 4,043	6,261 ± 1,569	0.004
Neutrophil count/μL	7,620 ± 3,673	3,928 ± 1,484	<0.001
Lymphocyte count/μL	921 ± 445	1,722 ± 782	<0.001
Platelet count, × 10^4^/μL	25.2 ± 10.2	18.0 ± 4.5	0.003
D-dimer, μg/mL	3.4 (2.7–7.7)	4.5 (1.7–6.6)	0.76
Cardiac function			
Ejection fraction (%)	61 ± 13	64 ± 8	0.31
Treatment modality and procedural findings, *n* (%)			
Balloon performed	18 (75.0)	15 (65.2)	0.46
Stent placement	8 (33.3)	4 (17.4)	0.32
Aspiration	23 (95.8)	20 (87.0)	0.35
Final below-the-ankle perfusion	22 (91.7)	19 (82.6)	0.42

SII, systemic immune-inflammation index; eGFR, estimated glomerular filtration rate; *n* (%), number (percentage).

There were no significant differences between the two groups regarding age or the prevalence of major comorbidities such as hypertension, diabetes mellitus, or atrial fibrillation. However, the proportion of males was significantly higher in the low SII group than in the high SII group (69.6% vs. 33.3%, *P* = 0.02). Regarding ischemia severity, patients in the high SII group exhibited a significantly higher prevalence of Rutherford class IIb ischemia (75.0% vs. 43.5%, *P* = 0.028) and combined severe ischemia, defined as Rutherford classes IIb and III (83.3% vs. 43.5%, *P* = 0.004). There was no significant difference between the groups in the prevalence of aorto-iliac involvement, a marker of proximal lesion location (Table 1).

In the additional *post hoc* exploratory analysis, SII values were significantly higher in patients with severe ischemia (Rutherford IIb–III) than in those without severe ischemia (Rutherford I–IIa) [median, 1426 [IQR, 674–2209] vs. 599 [IQR, 335–741]; *P* = 0.0067]. When patients were stratified by ischemia severity, the primary composite outcome occurred more frequently in the high-SII group than in the low-SII group both among patients without severe ischemia [2 of 4 [50.0%] vs. 2 of 13 [15.4%]] and among those with severe ischemia [13 of 20 [65.0%] vs. 3 of 10 [30.0%]]. However, these stratified comparisons were limited by the very small number of patients in each subgroup and were therefore interpreted as exploratory and descriptive.

Laboratory findings indicated that the high SII group had significantly higher white blood cell, neutrophil, and platelet counts, and significantly lower lymphocyte counts compared with the low SII group. Additionally, CRP levels were significantly higher in the high SII group, whereas serum albumin levels tended to be lower, although the difference was not statistically significant. In an additional exploratory analysis, SII showed a moderate positive correlation with CRP (Spearman's rho = 0.56, *P* = 0.0001; Supplementary Table 1). Serum creatinine and eGFR levels were comparable between the two groups, with no significant differences observed. There were no significant differences in treatment modalities, including balloon angioplasty, stent placement, or aspiration thrombectomy, between the two groups. Similarly, the use of medications at admission, such as aspirin, P2Y12 inhibitors, anticoagulants, and statins, did not differ significantly between the two groups. There was no significant difference between the groups in final below-the-ankle perfusion after endovascular treatment (Table 1).

### 3.2 Primary outcome

**Figure 2:**
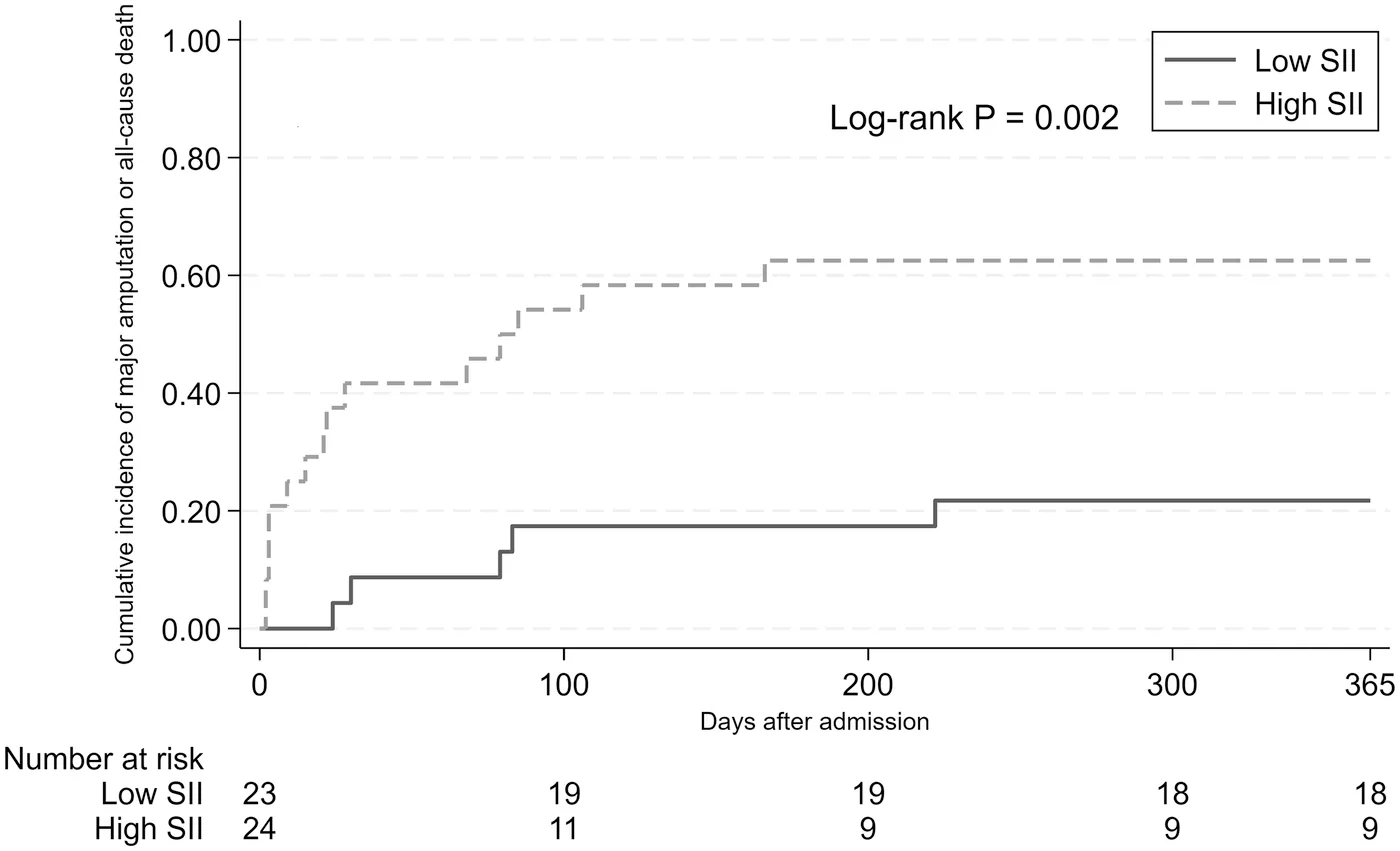
Kaplan–Meier curves showing the 12-month cumulative incidence of the primary composite outcome according to the systemic immune-inflammation index (SII) group. The event time was defined as the time to the first occurrence of either major amputation or all-cause death; patients without either event were censored at the last follow-up.

During the 12-month follow-up period, 20 patients (42.6%) experienced the composite primary outcome of major amputation or all-cause mortality. The incidence of the primary outcome was significantly higher in the high SII group (15 patients, 62.5%) than in the low SII group (5 patients, 21.7%) (*P* = 0.008; Table 2). Kaplan–Meier survival analysis revealed a significantly higher cumulative incidence of the primary outcome in the high SII group compared with the low SII group (log-rank test, *P* = 0.002; Figure 2).

**Table 2:** 12-month clinical outcomes stratified by median SII value.

Outcome, *n* (%)	High SII (*n* = 24)	Low SII (*n* = 23)	*P*-value
Primary composite outcome			
Major amputation or all-cause death	15 (62.5)	5 (21.7)	0.008
Secondary outcomes			
Major amputation	5 (20.8)	2 (8.7)	0.42
All-cause mortality	10 (41.7)	3 (13.0)	0.049

SII, systemic immune-inflammation index; *n* (%), number (percentage).

**Figure 3:**
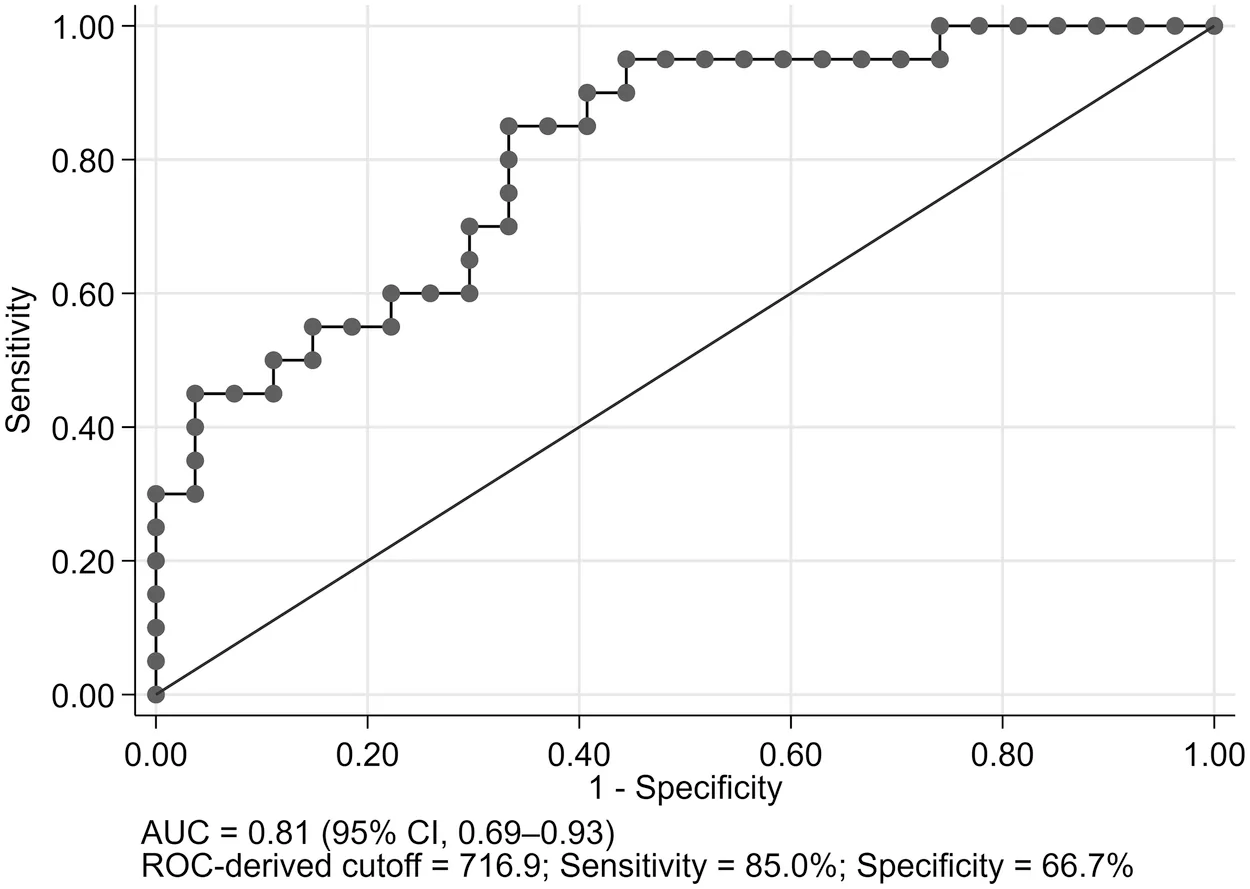
Receiver operating characteristic (ROC) curve analysis of the systemic immune-inflammation index (SII) for the primary composite outcome. The ROC-derived cutoff value of 716.9 was exploratory and should not be considered a clinically validated threshold. AUC, area under the curve; CI, confidence interval; SII, systemic immune-inflammation index.

ROC curve analysis showed an AUC of 0.81 for SII in relation to the primary composite outcome [95% confidence interval (CI), 0.69–0.93]. The ROC-derived cutoff value was 716.9, yielding a sensitivity of 85% and a specificity of 66.7% in this cohort (Figure 3). This cutoff should be interpreted as exploratory. In additional exploratory analyses, the AUCs for NLR, PLR, and CRP were 0.84 (95% CI, 0.72–0.95), 0.71 (95% CI, 0.55–0.86), and 0.78 (95% CI, 0.64–0.91), respectively. The overall comparison of AUCs among SII, NLR, PLR, and CRP did not show a statistically significant difference (*χ*^2^ = 6.35, *P* = 0.096; Supplementary Table 2).

### 3.3 Secondary outcomes

Major amputation occurred in seven patients (14.9%) overall—five (20.8%) in the high SII group and two (8.7%) in the low SII group. Although the incidence was higher in the high SII group, the difference was not statistically significant (*P* = 0.42). Major amputations occurred between 9 and 106 days after admission, with a median time to major amputation of 28 days (IQR, 19.5–49 days). All-cause mortality occurred in 13 patients (27.7%) and was significantly more frequent in the high SII group than in the low SII group [10 [41.7%] vs. 3 [13.0%], *P* = 0.049; Table 2]. Deaths occurred between 2 and 222 days after admission, with a median time to death of 22 days (IQR, 3–83 days). No patient experienced both major amputation and all-cause death during the 12-month follow-up period.

### 3.4 Multivariable analysis

In the exploratory Cox proportional hazards model adjusted for age, sex, severe ischemia (Rutherford IIb–III), and eGFR, older age [hazard ratio [HR], 1.12 per year; 95% confidence interval [CI], 1.04–1.20; *P* = 0.004] and high SII (HR, 4.40; 95% CI, 1.15–16.9; *P* = 0.031) were associated with an increased risk of the primary composite outcome (Table 3). However, the wide confidence interval for high SII indicates substantial statistical imprecision; therefore, this multivariable finding should be interpreted as exploratory and hypothesis-generating rather than confirmatory.

**Table 3:** Exploratory multivariable Cox model for the primary composite outcome.

Variable	HR	95% CI	*P*-value
Age (per year increase)	1.12	1.04–1.20	0.004
Male sex	1.98	0.74–5.26	0.17
Rutherford IIb–III	1.29	0.31–5.33	0.73
eGFR, mL/min/1.73 m^2^	1.004	0.99–1.02	0.66
High SII	4.40	1.15–16.9	0.031

SII, systemic immune-inflammation index; HR, hazard ratio; CI, confidence interval. This multivariable Cox model was exploratory and should be interpreted cautiously because of the limited number of outcome events and the wide confidence intervals.

When the SII was analyzed as a continuous variable in the same exploratory multivariable model, higher SII values were associated with the primary outcome (HR, 1.047 per 100-unit increase; 95% CI, 1.020–1.075; *P* < 0.001). Similarly, when the SII was dichotomized using the ROC-derived cutoff value, SII was also associated with the primary outcome (HR, 9.30; 95% CI, 2.09–41.40; *P* = 0.003). However, the wide confidence interval in the ROC-derived cutoff analysis indicates substantial statistical imprecision. Therefore, these sensitivity analyses should be interpreted as exploratory and do not establish a clinically actionable cutoff.

## 4 Discussion

This retrospective cohort study found that elevated baseline SII was associated with a higher incidence of the 12-month composite outcome of major amputation or all-cause mortality in patients undergoing endovascular therapy for ALI. In an exploratory multivariable Cox model adjusted for age, sex, ischemia severity, and renal function, high SII remained associated with the composite outcome; however, the estimate was imprecise, with a wide confidence interval. Given the small sample size and limited number of events, these findings should be considered hypothesis-generating and do not establish that the observed association is independent of ischemic injury or other clinical factors. To our knowledge, this is the first study to examine the association between SII and clinical outcomes specifically in patients with ALI undergoing endovascular therapy.

Interpretation of the observed association should account for several ALI-specific clinical and procedural factors that were unavailable or not included in the present analysis. Outcomes after endovascular treatment for ALI may be influenced by ischemia duration, embolic versus thrombotic etiology, thrombus burden, recurrent thrombosis or reintervention, fasciotomy, and postprocedural antithrombotic strategies. Although atrial fibrillation and baseline antithrombotic therapy were assessed as baseline characteristics, they were not included in the exploratory multivariable model because of the small sample size and limited number of outcome events. Residual confounding by these and other unmeasured ALI-specific factors therefore cannot be excluded, and the observed association between SII and adverse outcomes should not be interpreted as causal.

ALI is characterized by sudden arterial occlusion leading to rapid-onset ischemia often accompanied by an intense systemic inflammatory response (6). Despite recent advances in endovascular and surgical interventions (3–5), rates of mortality and limb loss remain high. Early and accurate risk stratification is therefore essential to improve outcomes; however, existing clinical and laboratory tools provide limited predictive precision. Conventional inflammatory markers such as CRP and leukocyte count provide only limited insight into the complex interplay among innate immunity, adaptive immunity, and thrombosis (12, 15). In the present exploratory analyses, SII showed discriminatory ability comparable to that of CRP and other inflammatory indices, although the study was not designed or sufficiently powered to establish superiority among biomarkers. Previous reports suggest that the SII is associated with clinical outcomes in various cardiovascular settings. For example, Morikawa et al. demonstrated that the SII was associated with adverse outcomes in patients undergoing carotid artery stenting (11). A recent meta-analysis further supported the prognostic relevance of the SII across various cardiovascular diseases, including coronary artery disease and heart failure (12). Given the limited data on the prognostic role of SII in ALI, our study focused on evaluating SII because it integrates multiple immune components and has shown consistent prognostic value in cardiovascular conditions.

The SII, a composite index derived from neutrophil, lymphocyte, and platelet counts, reflects both innate and adaptive immune activity. Neutrophils promote thrombosis and endothelial injury, partly through the formation of neutrophil extracellular traps (16, 17), whereas lymphopenia indicates immune suppression and is linked to poor outcomes in cardiovascular and systemic diseases (18, 19). Platelets further contribute to thromboinflammation and vascular damage, amplifying ischemic injury (20, 21). Thus, a high SII may identify patients with heightened inflammatory activation and impaired immune regulation, which may partly explain its observed association with adverse outcomes.

Other established inflammatory indices, such as the neutrophil-to-lymphocyte ratio and platelet-to-lymphocyte ratio, may also have prognostic value in vascular diseases. In the present exploratory ROC analyses, the AUC for SII was 0.81, whereas the AUCs for NLR, PLR, and CRP were 0.84, 0.71, and 0.78, respectively, with no statistically significant difference among these biomarkers. Therefore, the present findings do not demonstrate superiority or incremental prognostic value of SII over established inflammatory biomarkers. Rather, SII should be considered one of several inflammatory indices that may be associated with adverse outcomes in patients with acute limb ischemia undergoing endovascular therapy. Although the SII integrates platelet, neutrophil, and lymphocyte counts into a single composite index and may reflect multiple aspects of immune–inflammatory balance, whether it improves risk prediction beyond NLR, PLR, CRP, or established clinical variables requires validation in larger cohorts. Previous studies have also linked SII to coronary artery disease severity and functionally significant coronary stenosis (22, 23), supporting its relevance as a cardiovascular inflammatory marker, although these findings do not establish its incremental value in ALI.

In our study, patients with a high SII had more severe ischemia (Rutherford IIb–III) and higher CRP levels, consistent with greater inflammatory and ischemic burden at presentation. In a *post hoc* exploratory analysis, SII values were significantly higher in patients with severe ischemia than in those without severe ischemia, suggesting that elevated SII may partly reflect the extent of ischemic injury in ALI. Although the association between high SII and the primary composite outcome remained after adjustment for Rutherford IIb–III ischemia and renal function in the exploratory multivariable model, residual confounding by ischemia severity cannot be excluded, particularly given the small sample size and the broad categorization of ischemia severity. Accordingly, SII should be interpreted as a marker of the combined burden of ischemic injury, systemic inflammation, and immune dysregulation at presentation, rather than as a biomarker independent of ischemic injury.

The ROC curve analysis yielded an AUC of 0.81 for the primary composite outcome, with a ROC-derived cutoff value of 716.9 providing 85% sensitivity and 66.7% specificity in this cohort. Given the small sample size and single-center design, this cutoff should be regarded as exploratory and should not be considered a clinically validated threshold. Final below-the-ankle perfusion after endovascular treatment did not differ significantly between the SII groups, indicating that no clear group difference in this qualitative measure of distal reperfusion was observed. Nevertheless, because below-the-ankle perfusion was assessed qualitatively and SII is a nonspecific inflammatory index, these findings do not establish that the association between SII and adverse outcomes is independent of procedural reperfusion or specific to ALI-related inflammation. Elevated SII may instead reflect the combined systemic inflammatory, immune, and ischemic burden at presentation.

The present study did not evaluate whether SII-guided management improves clinical outcomes, and treatment strategies in this retrospective cohort were not determined according to SII levels. Therefore, elevated SII should not be used as a stand-alone determinant of revascularization strategy or antithrombotic therapy. In this context, SII may serve only as a potential adjunctive marker for identifying patients who warrant closer post-procedural surveillance and careful reassessment of limb perfusion. Whether SII adds prognostic information beyond established clinical factors and inflammatory biomarkers, or whether serial changes in SII after revascularization are clinically informative, should be evaluated in prospective studies.

In sensitivity analyses, similar associations were observed when SII was modeled as a continuous variable and when it was dichotomized using the ROC-derived cutoff value. However, the estimate based on the ROC-derived cutoff was highly imprecise, with a wide confidence interval. These analyses indicate that the findings were not entirely dependent on median-based categorization, but they neither establish the robustness of the association nor validate a clinically useful SII threshold. Larger independent cohorts are required to assess the reproducibility of these findings and any proposed cutoff value.

For secondary outcomes, major amputation was numerically more frequent in the high-SII group but did not differ significantly between groups, whereas all-cause mortality was significantly more frequent in the high-SII group. No patient experienced both major amputation and all-cause death during the 12-month follow-up period. These findings indicate that the association between SII and the primary composite outcome appears to have reflected all-cause mortality more strongly than major amputation. Accordingly, elevated SII should be interpreted as being associated with overall adverse prognosis after endovascular treatment for ALI, while its specific association with major amputation remains uncertain. Larger studies with sufficient numbers of limb events are needed to clarify the relationship between SII and limb-specific outcomes.

Previous studies have reported associations between SII and adverse outcomes across several cardiovascular and vascular conditions (11, 12, 23–25). However, a recent meta-analysis of lower extremity arterial disease found no significant association between SII and restenosis, whereas NLR showed more consistent associations with mortality, amputation, and amputation-free survival (26). Because restenosis primarily reflects chronic vascular remodeling after revascularization, these findings may not be directly applicable to the acute thrombotic and inflammatory processes of ALI. Nevertheless, they reinforce the need to avoid assuming that SII is superior to other inflammatory indices. Given the limited evidence in ALI, the present findings should be regarded as preliminary and require validation in larger multicenter cohorts across different treatment strategies and patient populations.

In addition, prior registry-based studies have identified renal dysfunction, ischemia severity, and older age as important prognostic factors in ALI (14, 27). In the present exploratory analysis, the association between SII and the 12-month composite outcome remained after adjustment for age, sex, ischemia severity, and renal function. However, given the limited sample size, wide confidence intervals, and lack of demonstrated incremental value over established inflammatory biomarkers or clinical risk models, this finding should be interpreted as hypothesis-generating. The potential contribution of SII to risk stratification beyond conventional clinical factors and established inflammatory biomarkers therefore requires further validation.

### 4.1 Limitations

This study has several limitations. First, its single-center, retrospective design and the inclusion of an elderly Japanese cohort may limit the generalizability of the findings to younger patients, other ethnic and geographic populations, and healthcare systems with different baseline risk profiles, referral patterns, revascularization strategies, and post-procedural care. Validation in larger multicenter cohorts representing more diverse patient populations and clinical settings is therefore required. Furthermore, because the study period extended from 2013 to 2024, endovascular devices, procedural techniques, antithrombotic strategies, and post-procedural care may have changed over time. These temporal changes were not systematically captured or adjusted for because of the limited sample size and may have influenced clinical outcomes. The small sample size may have limited statistical power, particularly for the individual secondary outcome of major amputation. Moreover, the limited number of primary outcome events may have reduced the stability and precision of the multivariable Cox model estimates, as reflected by the wide confidence intervals. Although the number of covariates was intentionally restricted to reduce the risk of overfitting, model instability cannot be excluded. Accordingly, the estimated hazard ratios should be interpreted cautiously and regarded as exploratory rather than stable or confirmatory estimates, and these findings require validation in larger multicenter cohorts.

Because major amputation and all-cause mortality are clinically distinct outcomes with different pathophysiological mechanisms, the composite endpoint should be interpreted cautiously despite its common use in studies of ALI. Major amputation was numerically more frequent in the high-SII group but did not differ significantly between groups, suggesting that the association with the composite endpoint may have reflected all-cause mortality more strongly than limb-specific events. Nevertheless, a limb-specific association cannot be excluded because of the small number of amputations. All-cause death may also have acted as a competing risk for major amputation; however, formal competing-risk analysis was not performed because of the small number of major amputation events. All-cause rather than cardiovascular mortality was used because cause-specific mortality could not be reliably adjudicated in this retrospective study, and the inclusion of non-vascular deaths may have attenuated vascular-specific associations. In addition, follow-up information for some patients was obtained through structured telephone interviews rather than direct clinical assessment, which may have introduced differential outcome ascertainment. Larger multicenter studies with sufficient numbers of limb-specific events are needed to confirm these findings.

The ROC-derived cutoff value of 716.9 requires external validation before its reproducibility or clinical relevance can be established across different patient populations and healthcare settings. In addition, only admission SII values were available in this study; therefore, we could not determine whether serial changes in SII after revascularization were associated with clinical outcomes beyond a single baseline measurement. Future prospective studies should evaluate whether longitudinal SII assessment reflects changes in thromboinflammatory activity or treatment response. Moreover, several ALI-specific clinical and procedural variables that may influence outcomes were not consistently available, including precise ischemia duration, embolic versus thrombotic etiology, quantitative thrombus burden, recurrent thrombosis or reintervention, fasciotomy, and detailed longitudinal antithrombotic strategies. Although atrial fibrillation and baseline antithrombotic therapy were assessed as baseline characteristics, these variables were not included in the exploratory multivariable model because of the limited sample size and number of outcome events. Lesion location was assessed angiographically and categorized according to the presence or absence of aorto-iliac involvement; however, more detailed lesion-level analyses were not performed. Although final below-the-ankle perfusion after endovascular treatment was comparable between the groups, this variable was assessed qualitatively as a binary angiographic finding based on completion angiography, and no standardized quantitative perfusion score or independent core laboratory assessment was used. Therefore, subtle differences in distal runoff quality, microvascular perfusion, procedural factors, or postprocedural antithrombotic management may not have been fully captured and may represent sources of residual confounding.

Although patients with major inflammatory confounders, including active infection, active malignancy, and systemic inflammatory disease, were excluded, occult inflammatory conditions or unrecognized comorbid inflammatory processes cannot be completely ruled out. Therefore, elevated SII should be interpreted as a marker of systemic inflammatory and immune status at presentation rather than as a biomarker specific to ALI-related inflammation. CRP was not included in the primary multivariable Cox model because of the limited number of outcome events and the potential overlap between inflammatory biomarkers, which could have increased the risk of overfitting and model instability. Although we performed additional exploratory comparisons of SII with NLR, PLR, and CRP, the limited sample size precluded a definitive assessment of whether SII provides incremental value beyond these established inflammatory markers for risk stratification. Future studies with larger sample sizes should directly compare SII, NLR, PLR, CRP, and other inflammatory biomarkers and evaluate whether these markers improve established clinical risk models in patients with acute limb ischemia.

### 4.2 Conclusions

This exploratory retrospective cohort study showed that an elevated baseline SII was associated with a higher risk of the composite outcome of major amputation or all-cause mortality in patients with ALI undergoing endovascular therapy. This association appeared to reflect overall adverse prognosis, with the composite outcome substantially influenced by all-cause mortality, whereas the specific association between SII and major amputation remains uncertain.

Exploratory comparisons did not demonstrate superiority of SII over NLR, PLR, or CRP. Because the SII is derived from routine hematologic parameters, it may represent a simple adjunctive marker for risk stratification in this high-risk population. However, the present findings, including the ROC-derived cutoff analysis, should be considered hypothesis-generating and require validation in larger prospective multicenter studies incorporating longitudinal biomarker assessment, detailed ALI-specific clinical and procedural variables, and sufficient numbers of limb-specific events.

## Data Availability

The datasets presented in this article are not readily available because they contain patient-level clinical data and are subject to patient privacy concerns and institutional regulations. De-identified data may be made available from the corresponding author upon reasonable request, subject to Institutional Review Board approval and appropriate data-sharing agreements. Requests to access the datasets should be directed to Shuji Morikawa, MD, PhD Department of Cardiology, Chutoen General Medical Center, morinko1@hotmail.co.jp.
