# Supplementary material for: Prognostic value of the systemic immune-inflammation index in acute limb ischemia undergoing endovascular therapy
Source: Front Cardiovasc Med. 2026 Aug 12;13:1732152. doi: 10.3389/fcvm.2026.1732152 (PMC13506949; doi:10.3389/fcvm.2026.1732152)
Supplement: Supplementary file 1 [file Table1.docx]

**Supplementary Table 1. Correlation between SII and CRP**

| **Variables** | **Spearman’s rho** | **P-value** |
| --- | --- | --- |
| SII and CRP | 0.56 | 0.0001 |

CRP, C-reactive protein; SII, systemic immune-inflammation index.

**Supplementary Table 2. Discriminatory ability of inflammatory biomarkers for the primary composite outcome**

| **Biomarker** | **AUC** | **95% CI** |
| --- | --- | --- |
| SII | 0.81 | 0.69–0.93 |
| NLR | 0.84 | 0.72–0.95 |
| PLR | 0.71 | 0.55–0.86 |
| CRP | 0.78 | 0.64–0.91 |

AUC, area under the curve; CI, confidence interval; CRP, C-reactive protein; NLR, neutrophil-to-lymphocyte ratio; PLR, platelet-to-lymphocyte ratio; SII, systemic immune-inflammation index.

The overall comparison of AUCs among SII, NLR, PLR, and CRP did not show a statistically significant difference (χ² = 6.35, P = 0.096).
